# A Cross-Lagged Panel Analysis of Psychometric Intelligence and Achievement in Reading and Math

**DOI:** 10.3390/jintelligence5030031

**Published:** 2017-09-01

**Authors:** Marley W. Watkins, Kara M. Styck

**Affiliations:** 1 Department of Educational Psychology, Baylor University, Waco, TX 76798, USA; 2 Department of Educational Psychology, The University of Texas at San Antonio, San Antonio, TX 78207, USA; kara.styck@utsa.edu

**Keywords:** intelligence, achievement, WISC, cross-lag, structural equation modeling (SEM)

## Abstract

A cross-lagged panel analysis of Wechsler Intelligence Scale for Children-Fourth Edition (WISC-IV) intelligence test scores and reading and math achievement test scores of 337 students twice assessed for special education eligibility across a test-retest interval of 2.85 years was conducted. General intelligence (*g*) was loaded by the four WISC-IV factor index scores whereas reading and math were composite scores. After confirming measurement invariance, it was found that *g*, reading, and math were stable across time and synchronously correlated. The cross-lagged paths from *g* at time 1 to reading and math at time 2 (0.26 and 0.39, respectively) were both significantly greater than zero whereas the paths from reading and math at time 1 to *g* at time 2 (0.03 and 0.23, respectively) were not statistically significant. Given this pattern of relationships and extant research on the correlates of general intelligence, it was tentatively inferred that general intelligence was the temporal precursor to reading and math achievement.

## 1. Introduction

The relationship between psychometric intelligence and academic achievement is disputed: some researchers consider the two measures to be similar if not identical [1], others assert that intelligence test scores and academic achievement test scores mutually influence each other [2], and still others hold that intelligence is causally related to achievement [3]. For example, Ceci [1,4,5] asserted that measured intelligence merely reflects direct and indirect school learning. The two constructs are analogous from this perspective. Ceci [1] argued that the robust positive correlation between the highest completed grade in school and measured intelligence (even after controlling for timing of school entry, socioeconomic status, and other social variables) provides evidence in support of his position. Likewise, it has been documented that intelligence test scores tend to regress during summer break, share a positive correlation with school attendance, and are adversely impacted by delays in the onset of school or early school termination. These and other factors have been cited as further evidence that school substantially influences measured intelligence [1,5].

A second hypothesis that has been posited is that measured intelligence and achievement mutually influence each other and are bidirectional as a result [2]. Ferrer et al. [6] applied linear dynamic models to a longitudinal dataset containing 445 children who were annually assessed from first through twelfth grades. Results showed a positive and bidirectional relationship between measured intelligence and reading achievement. Moreover, the magnitude of this relationship diminished over time. The bidirectional influence of measured intelligence and achievement was strongest during first through third grades, and weakest from ninth through twelfth grades. More recently, Ferrer, Shaywitz, Holahan, Marchione, and Shaywitz [7] used the same dataset to investigate the degree to which the dynamic relationship between measured intelligence and achievement differed for typical readers and readers diagnosed with dyslexia. Results indicated a bidirectional relationship between intelligence test scores and achievement test scores for typical readers and small to negligible influences of measured intelligence on reading achievement and reading achievement on measured intelligence, respectively, for readers diagnosed with dyslexia. Consequently, Ferrer et al. [7] concluded that, “dyslexic readers are characterized by a disruption in the interconnection between IQ and reading over time” (p. 99).

The final hypothesis is that psychometric intelligence shares a causal linkage with academic achievement [3]. Soares, Lemos, Primi, and Almeida [8] recently explored this notion in a longitudinal study of 284 Portuguese middle school students. They reported that class grades at the end of seventh grade mediated the relationship between general intelligence test scores and class grades at the end of ninth grade. This led the researchers to conclude that “it is important to address the prediction of academic achievement as a possible twofold equation: intelligence and prior level of knowledge” ([8], p. 78). Reynolds and Turek [9] drew similar conclusions. They examined the relationship between verbal comprehension-knowledge (Gc), a broad cognitive ability defined in the Cattell–Horn–Carroll (CHC) theory of intelligence [10,11,12], and reading comprehension in a longitudinal study of 1079 children who were assessed around ages 9, 11, and 15. Results indicated a unidirectional relationship wherein Gc scores predicted reading comprehension scores, but the reverse was found to be untrue. Moreover, Reynolds et al. [9] noted that socioeconomic status and indicators of prior achievement (i.e., third grade sight words and relative reading volume) exhibited positive effects on the relationship between the two variables. Higher Gc and reading comprehension scores were observed in third grade for children with higher Gc scores at age two. In addition, children with higher Gc scores at age two exhibited accelerated growth on measures of Gc and reading comprehension over time. Quinn, Wagner, Petscher, and Lopez [13] replicated these results in a longitudinal study with an independent sample of 316 first grade students who were assessed annually through fourth grade, which led them to conclude that, “the present results support Anderson and Freebody’s (1981) instrumentalist hypothesis that vocabulary knowledge has a causal influence on reading comprehension” (p. 171).

The degree to which measured intelligence predicts mathematics achievement has also been explored, though to a lesser extent. Primi, Ferrao, and Almeida [14] evaluated the relationship between fluid intelligence (Gf) and mathematics achievement in a longitudinal study of 166 middle school students assessed at the beginning and end of seventh and eighth grades. Results indicated that fluid intelligence (Gf) test scores predicted mathematics test scores over time. Primi et al. [14] also noted that students with higher initial Gf scores exhibited steeper growth in mathematics test scores over a two-year period compared to students with lower initial Gf scores, thereby echoing results of research investigating the predictive relationship between measured intelligence and reading achievement [8,9,13].

**Figure 1:**
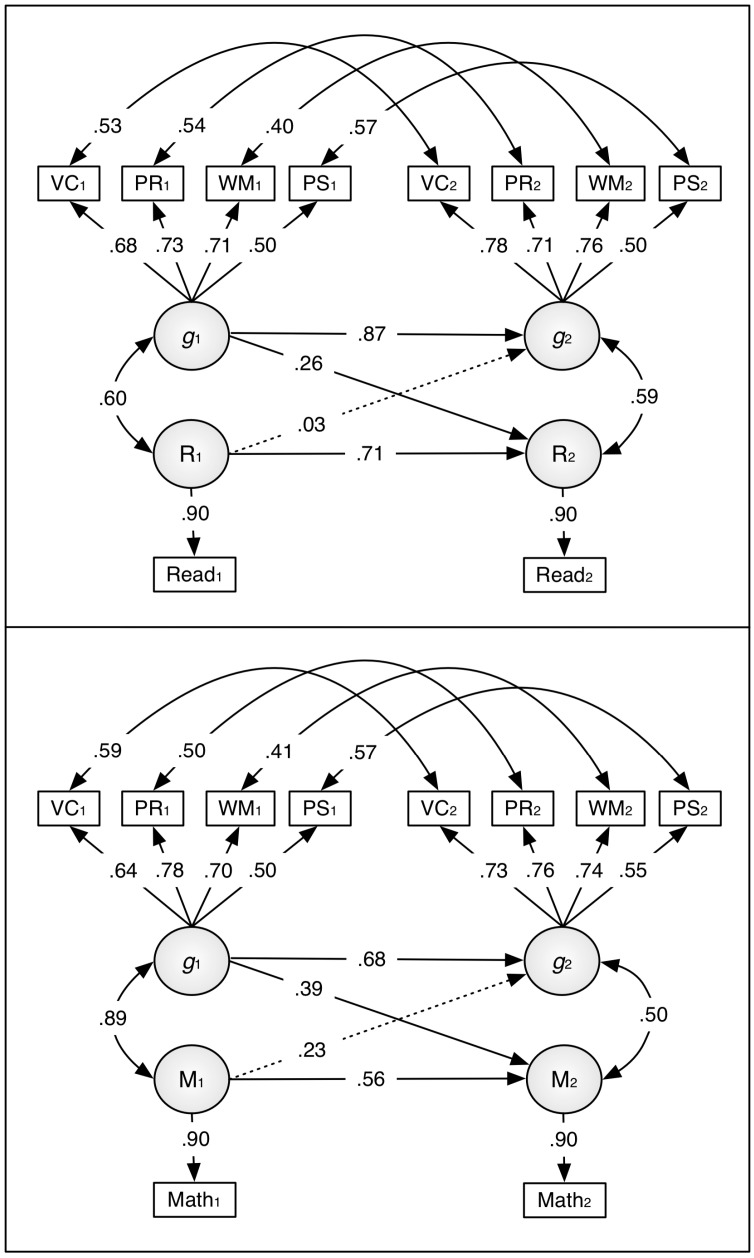
Cross-lagged panel model of intelligence measured by the Wechsler Intelligence Scale for Children-Fourth Edition (WISC-IV) and achievement in reading (upper panel) and math (lower panel) for 337 students twice tested for special education eligibility across a 2.85 year interval. Note: VC = Verbal Comprehension Index, PR = Perceptual Reasoning Index, WM = Working Memory Index, PS = Processing Speed Index, *g* = General Intelligence, R = reading factor, Read = reading composite score, M = math factor, and Math = mathematics composite score. Numerical subscript indicates test (time 1) or retest (time 2) occasion. Standardized coefficients with error/disturbance terms omitted for visual clarity. Nonsignificant paths are dotted.

Nevertheless, the dispute regarding the relationship between intelligence and academic achievement can only be resolved with evidence from experimental studies that are not possible to implement. Consequently, cross-lagged panel correlation designs where both intelligence and achievement tests are repeated across time have been suggested as quasi-experimental alternatives [15]. A cross-lagged panel correlation model includes all possible correlations of intelligence and achievement at two or more time points. The correlations between intelligence and achievement scores at each time point are synchronous correlations (measures of concurrent validity), the correlations between intelligence and achievement across time are autocorrelations (measures of stability), and the correlations of intelligence and achievement with each other at different points in time are cross-lags. The main focus is on the cross-lagged correlations, because a variable that is consistently followed by change in another variable satisfies the time precedence and covariation conditions required for causal inference [16,17]. Therefore, if intelligence causes achievement then the cross-lagged correlation from intelligence at time 1 to achievement at time 2 would be larger than the cross-lagged correlation from achievement at time 1 to intelligence at time 2 (see Figure 1 for an illustration). Other patterns of cross-lagged correlations could result if achievement causes intelligence or intelligence and achievement are caused by some other variable or they mutually influence each other.

The first implementation of a cross-lagged panel correlation design to investigate the causal preponderance of intelligence and achievement employed group tests with a large sample of students [18]. Results indicated that intelligence was predominant for suburban students but not for urban students but both groups exhibited substantial cross-lagged correlations, suggesting the possibility of mutual influence. Mutual influence was also found in a subsequent cross-lagged panel analysis with disadvantaged preschoolers [19]. These results were contested by Rogosa [20], who contended that the cross-lagged panel correlation model “does not provide sound information about causal effects” because it lacks “an explicit definition of a causal effect” (p. 246) and because the size of the cross-lagged correlations are confounded by the differential stabilities of the variables and the synchronous correlation at time 1 [16].

Rogosa [20] suggested that a regression/path model for longitudinal panel data might be more plausible because it controls for initial values but acknowledged that “methods for detecting patterns of causal influence from panel data are far from fully developed” (p. 257). Nevertheless, a structural path model using a cross-lagged monozygotic-differences (MZ) design to study the longitudinal genetic relationship between intelligence and reading achievement among 1890 twin pairs found that reading differences at several ages were significantly related to later intelligence differences [21]. The MZ design is considered “among the best methods to achieve strong internal validity because it controls for a wide range of confounding factors, either genetic or environmental” ([22], p. 376). However, the measurement of intelligence included only selected subtests (four at age 7 years, two at age 16 years) administered either by telephone or via the internet. Reading achievement was also assessed via telephone or internet. At age 7 years, reading was assessed with a reading fluency measure and teacher ratings whereas at age 16 years it was assessed with two comprehension measures. It is not clear why measured variables were preferred over latent variables in this study because path models are substantially affected by measurement error that makes their use questionable with measured variables [23]; nor why anything other than small effects would be expected from an MZ design employed with intelligence and reading measures that are both heavily influenced by genetic factors [24,25].

Further development of cross-lagged panel models occurred within a structural equation modeling (SEM) framework that combines multiple regression and factor analysis [26,27]. SEM allows an assessment of the cross-construct relationships after controlling for within-construct relationships and analysis of latent variables after removing the biasing effect of measurement error on autoregressive and cross-lagged estimates [26,28,29,30]. Within this model, “if the cross-lagged effect is significant in one direction but not the other, findings are consistent with the hypothesis that the causal effect works in one direction but not the other” ([31], p. 123).

Given these attributes, cross-lagged panel models in an SEM framework have been recommended as superior to path models [26,31,32] but have infrequently been used to investigate the causal linkage between intelligence and achievement. The only extant study is an analysis of cross-lagged Wechsler Intelligence Scale for Children-Third Edition (WISC-III; [33]) first-order verbal and perceptual organization factors and achievement in reading and math for 289 students twice evaluated for special education eligibility [34]. That study found that intelligence was predictive of future achievement whereas achievement was not predictive of future intelligence. Given the paucity of research on the relationship between psychometric intelligence and academic achievement using cross-lagged panel models, the current study applied a cross-lagged panel model within a SEM framework to individually administered tests of intelligence and achievement to elucidate the causal precedence of ability and achievement. Moreover, the present study expands upon prior cross-lagged panel analyses of psychometric intelligence and achievement, because psychometric *g* has not been previously included in cross-lagged panel models [34].

## 2. Method

*Participants.* Following approval of university institutional review board (IRB) and school district authorities, approximately 7500 special education files in two school districts located in the Southwestern United States were reviewed and 337 cases were identified that included Wechsler Intelligence Scale for Children-Fourth Edition (WISC-IV; [35]) factor index scores, a composite (total) reading score, and a composite (total) math score from two longitudinal assessments conducted in the years 2003 through 2010. The majority of the participants were male (69%) with a mean age of 8.7 (*SD* = 1.5) years at first testing and 11.6 (*SD* = 1.7) years at second testing for an average test-retest interval of 2.85 (*SD* = 0.6) years. The reported ethnic background of sample participants was 80% White, 11% Hispanic, 7% Black, and 2.0% other. Participants’ special education diagnoses were determined by school district multidisciplinary teams to be 66% learning disabled, 7% attention-deficit/hyperactivity disorder (ADHD), 8% emotionally disabled, 6% non-handicapped, 5% autism, 2% intellectually disabled, 3% language impaired, 2% health impaired, and 1% other. No other demographic information was collected to respect the privacy of the participants and to meet the requirements of cooperating school districts.

### 2.1. Measures

#### 2.1.1. Intelligence

*WISC-IV.* The WISC-IV [35] is an individually administered intelligence test for individuals between the ages of 6 and 16 years normed with a nationally representative sample of 2200 children and adolescents. The WISC-IV contains 15 subtests, 10 core and 5 supplemental, each with a mean of 10 and a standard deviation of 3. The 10 core subtests are used to form a Full Scale Intelligence Quotient (FSIQ) score as well as four index scores with a mean of 100 and a standard deviation of 15: Verbal Comprehension Index (VCI), Perceptual Reasoning Index (PRI), Working Memory Index (WMI), and Processing Speed Index (PSI). The VCI is based on the Similarities, Vocabulary, and Comprehension subtests and is thought to measure verbal concept formation. The PRI is based on the Block Design, Matrix Reasoning, and Picture Concepts subtests and is thought to measure non-verbal and fluid reasoning. The WMI is based on the Digit Span and Letter-Number Sequencing subtests and is thought to measure working memory. The PSI is based on the Coding and Symbol Search subtests and is thought to measure speed of information processing. Considerable evidence regarding reliability and validity of WISC-IV scores was reported by its publisher [36] and psychometric evidence from clinical samples has been supportive [37,38,39].

#### 2.1.2. Achievement

Achievement was measured by multiple instruments, but the majority of the participants were administered contemporary versions of the Wechsler Individual Achievement Test (WIAT) and the Woodcock-Johnson Tests of Achievement (WJ-Ach). Approximately 23% of the participants were administered a WIAT at both assessment occasions, 33% a WJ-Ach at both test and retest, 19% a WIAT at test and WJ-Ach at retest, and 22% a WJ-Ach at test and WIAT at retest. Although special education records usually included composite achievement scores, they inconsistently included academic subtest scores. Consequently, composite achievement scores were employed because of their uniform availability and breadth of coverage. Composite reading and mathematics scores (*M* = 100, *SD* = 15) from the WIAT (i.e., the Reading and Mathematics composite scores) and WJ-Ach (i.e., the Broad Reading composite score and the Broad Math composite score) have both evidenced robust reliability and validity evidence [40] and highly correlate (~0.85) across revisions and with each other [41,42].

*WJ-Ach.* The WJ-Ach is an individually administered achievement test for individuals aged 2–90 years normed with a nationally representative sample of 8818 individuals including undergraduate and graduate college students. It contains 22 subtests intended to measure reading, mathematics, writing, oral language, and academic knowledge. The standard battery includes 12 subtests that can be combined to form 8 composite scores: Total Achievement, Oral Language, Broad Reading, Broad Math, Broad Written Language, Academic Skills, Academic Fluency, and Academic Applications. The extended battery includes 10 additional subtests that can be combined with standard battery subtests to form 10 additional composite scores: Oral Language-Extended, Oral Expression, Listening Comprehension, Basic Reading Skills, Reading Comprehension, Math Calculation Skills, Math Reasoning, Basic Writing Skills, Written Expression, and Phoneme/Grapheme Knowledge. All WJ-Ach subtest and composite scores have a mean of 100 and a standard deviation of 15. 

*WIAT.* The WIAT is an individually administered test of achievement for individuals aged four through adulthood. It was normed with a nationally representative sample of individuals aged 4–85 and includes age-based (*n* = 2950), grade-based (*n* = 3600), college grade-based (*n* = 707), and adult age-based norms (*n* = 515). The WIAT contains 9 subtests intended to measure oral language, reading, written language, and mathematics that can be combined to form 4 composite scores: Reading, Mathematics, Written Language and Oral Language. WIAT subtest and composite scores have a mean of 100 and a standard deviation of 15. 

### 2.2. Analyses

Analyses were conducted with Mplus 8.0 for the Macintosh [43] and its full information maximum likelihood (FIML) methods were used to account for the small proportion of missing data points (<1%). The data were significantly multivariately skewed (χ^2^ (364) = 1836.9, *p* < 0.001) and kurtotic (χ^2^ (1) = 205.9, *p* < 0.001) so model estimation employed the MLR robust maximum likelihood estimator. Latent variables were scaled by fixing a reference indicator, the Mplus default. Model fit was considered acceptable if comparative fit index (CFI) ≥ 0.90 and root mean square error of approximation (RMSEA) < 0.08 and deemed good if CFI ≥ 0.95 and RMSEA ≤ 0.06 [44].

The cross-lagged panel model is illustrated in Figure 1 [30]. The general intelligence factor at each testing occasion was formed from the four factor index scores of the WISC-IV. As a prerequisite to cross-lagged analyses, the invariance of this WISC-IV structure across time was tested and found to exhibit configural, metric, and scalar invariance. The reading and math achievement constructs were created by fixing the error of their composite variables on the basis of their variance and a conservatively estimated reliability of 0.80 rather than relying on factors with only two indicators [26] or manifest variables [23]. Given their repeated administration, WISC-IV factor score errors were allowed to correlate across time but the reading and math construct errors could not be correlated across time because they were fixed [44].

## 3. Results

Descriptive statistics for the WISC-IV and achievement scores across test and retest occasions are presented in Table 1. Although lower than the normative samples, IQ and achievement scores were consistent with other samples of referral students [34,37]. Parameter estimates did not differ by more than 0.01 between FIML estimates and listwise deletion so FIML estimates are reported.

The cross-lagged model for reading achievement (upper panel of Figure 1) exhibited good fit to the data with χ^2^ (27) = 47.5, *p* = 0.009, RMSEA = 0.047 (90% confidence interval (CI), 0.024–0.069), and CFI = 0.988. The model explained 79% of the variance in both general intelligence and reading achievement at time 2 and the standardized root mean square residual (SRMR) of 0.035 indicated that average residuals were small. With the exception of the cross-lagged path from reading achievement at time 1 to general intelligence at time 2, all of the paths were statistically significant. Both general intelligence and reading were stable across time (0.87 and 0.71, respectively) and the synchronous correlations between general intelligence and reading were both strong (0.60 and 0.59, respectively). The pattern of significant (intelligence to reading of 0.26) and non-significant (reading to intelligence of 0.03) cross-lagged paths allows the inference of temporal precedence of general intelligence to reading achievement.

The cross-lagged model for math achievement (lower panel of Figure 1) also exhibited good fit to the data with χ^2^ (27) = 40.7, *p* = 0.044, RMSEA = 0.039 (90% CI, 0.006–0.063), and CFI = 0.993. The model explained 79% of the variance in general intelligence and 86% of the variance in math achievement at time 2 and the SRMR of 0.023 indicated that residuals, on average, were small. All of the paths were statistically significant except the cross-lagged path from math achievement at time 1 to general intelligence at time 2. General intelligence and math were both stable across time (0.68 and 0.56, respectively) and the synchronous correlations between general intelligence and math were robust (0.89 vs. 0.50, respectively). The pattern of significant (intelligence to math of 0.39) vs. non-significant (math to intelligence of 0.23) cross-lagged paths allows the inference of temporal precedence of general intelligence to math achievement. However, the path of 0.23 from math at time 1 to intelligence at time 2 is substantial and could attain statistical significance with a larger sample, admitting the possibility of mutual influence.

## 4. Discussion

Results indicated that intelligence at time 1 predicted reading achievement at time 2, whereas reading achievement at time 1 did not predict intelligence at time 2. Consequently, it appears that intelligence precedes reading achievement in time. Both of these conditions are necessary for drawing causal inferences. Similar results were found for the relationship between intelligence and math achievement, albeit less distinctively. Moreover, the magnitude of the path from math achievement at time 1 to intelligence at time 2 suggests that measured intelligence and math achievement may share a bidirectional relationship. CHC theory posits that intelligence is a multidimensional construct composed of broad and narrow abilities [10,11,12]. One such broad ability is quantitative knowledge. It is possible that quantitative knowledge exerts some influence to explain the relationship between measured intelligence and math achievement in the present sample. There is also some evidence to suggest that working memory plays a distinct role in the prediction of academic achievement [45,46]. However, the degree to which broad abilities such as quantitative knowledge and working memory offer incremental validity beyond general intelligence in predicting academic achievement is disputed within the literature [39,47].

These results are consistent with those reported by Watkins et al. [34] with a similar sample of students twice evaluated for special education eligibility. Unfortunately, both studies included only two measurement waves and both samples were vulnerable to selective attrition because only students who were reevaluated were included. Three or more measurement waves would have allowed a more powerful design [48] and random sampling would have enhanced generalizability. Unfortunately, neither cross-lagged panel study could rule out the possibility that the observed relationships were spurious [16]. Notwithstanding limitations, cross-lagged panel studies are the only modern investigations of the causal precedence of ability and achievement within a SEM framework. These consistent results help “in building an argument for a causal effect” ([30], p. 271) of intelligence on academic achievement.

Additional support for the causal intelligence-achievement relationship argument has been provided by genetic research that has shown general intelligence to be highly heritable with moderate overlap between the genes responsible for intelligence and those responsible for reading and math achievement [25]. Further, general intelligence has been shown to be positively related to self-control, academic achievement, mental health, physical health, job performance, employment rate, and lifespan (e.g., [3,49,50,51,52,53,54,55,56,57,58,59]). Extended discussions of these relationships have been provided by Machintosh [60], Jensen [3], and Cooper [50]. However, causality has been debated by philosophers and scientists for centuries [61]. Cross-lagged panel studies satisfy the time precedence and covariation requirements of causal inference as previously stated. A third requirement for determining causality posited by philosopher John Stuart Mill as cited in Shadish, Cook, and Campbell [62] is the degree to which other plausible explanations exist to explain the relationship between the two variables thought to share a causal linkage. It is possible that variables such as gender, socio-economic status, or the richness of the home learning environment may explain the relationship between psychometric intelligence and achievement. Sternberg, Gigorenko, and Bundy [63] argue that, “… it may not be IQ itself that is responsible for these effects, but rather the encouragement or opportunities given to individuals with high IQ” (p. 7). In a longitudinal study of 205 children assessed annually from approximately 4–23 years old, Schneider, Niklas, and Schmiedeler [64] reported that stability of intelligence test scores was higher for participants with lower initial intelligence test scores compared to participants with higher initial intelligence test scores. Furthermore, Schneider et al. [64] reported that intelligence test scores at age seven predicted progress in compulsory education and college graduation. The extant literature categorically supports an educational advantage for children with high measured intelligence. There is ample evidence to support psychometric intelligence as an important predictor of academic achievement [3,8,9,13], and that it does not act alone [63]. Unfortunately, information regarding potentially relevant third variables was unavailable for the sample analyzed in the present study and their absence in the models tested represents a limitation. 

Similar to prior studies with the WISC [65,66], the synchronous correlations between measured intelligence scores and math achievement scores were stronger than the synchronous correlations between measured intelligence scores and reading achievement scores. However, we found that the magnitude of the cross-lagged relationship between measured intelligence and reading achievement was stronger than the cross-lagged relationship between measured intelligence and math achievement. These findings correspond to those of Schneider and Niklas [67] who reported that math achievement was better predicted by psychometric intelligence and prior math skills, whereas reading achievement was better predicted by psychometric intelligence alone in a longitudinal study of 205 children who were assessed at ages 6, 8, 10, 18, and 23. However, their model included reading decoding speed assessed at age 8 as the only precursor to reading achievement assessed at age 23 and the researchers noted this as a possible explanation for the results. Alternatively, these results might be related to this sample of students enrolled in special education given that one diagnostic marker for special education eligibility is impaired academic achievement [68].

In either case, causality is a question of formidable complexity [17,69], and it will require powerful new methodology or technology for satisfactory resolution of the IQ-achievement relationship [70,71,72]. Until then, it seems reasonable to tentatively conclude that intelligence is a temporal precursor to reading and math achievement or to allow the possibility of reciprocal influence.

## Figures and Tables

**Table 1:** Correlations and Descriptive Statistics for WISC-IV Index Scores and Reading Composite Scores from 337 Students Twice Tested for Special Education Eligibility across a 2.85 Year Interval.

	VCI_1_	PRI_1_	WMI_1_	PSI_1_	R_1_	M_1_	VCI_2_	PRI_2_	WMI_2_	PSI_2_	R_2_	M_2_
VCI_1_	1.00											
PRI_1_	0.48	1.00										
WMI_1_	0.46	0.55	1.00									
PSI_1_	0.31	0.38	0.39	1.00								
R_1_	0.41	0.35	0.42	0.22	1.00							
M_1_	0.52	0.63	0.56	0.38	0.48	1.00						
VCI_2_	0.72	0.52	0.42	0.27	0.43	0.56	1.00					
PRI_2_	0.43	0.73	0.46	0.40	0.32	0.56	0.59	1.00				
WMI_2_	0.49	0.52	0.65	0.34	0.39	0.55	0.56	0.54	1.00			
PSI_2_	0.27	0.34	0.33	0.64	0.15	0.41	0.34	0.43	0.39	1.00		
R_2_	0.48	0.41	0.44	0.25	0.69	0.49	0.59	0.42	0.52	0.27	1.00	
M_2_	0.51	0.63	0.60	0.50	0.42	0.73	0.57	0.60	0.60	0.50	0.56	1.00
*M*	93.4	96.1	89.3	91.9	84.6	89.6	93.3	96.0	88.6	90.0	87.5	88.1
*SD*	12.1	14.4	12.7	15.2	12.9	13.0	12.8	14.7	14.3	15.1	12.9	15.5

Note: VCI is Verbal Comprehension Index; PRI is Perceptual Reasoning Index; WMI is Working Memory Index; PSI is Processing Speed Index; R is the Reading Composite score; M is the math composite score; subscript 1 represents values at time 1; subscript 2 represents values at time 2; *M* is the mean and *SD* is the standard deviation.

## Supplementary Materials

Supplementary File 1

## References

[1] Ceci S.J. (1991). How much does schooling influence general intelligence and its cognitive components? A reassessment of the evidence. Dev. Psychol..

[2] Mayer R.E., Sternberg R.J., Kaufman S.B. (2011). Intelligence and achievement. Cambridge Handbook of Intelligence.

[3] Jensen A.R. (1998). The G Factor: The Science of Mental Ability.

[4] Ceci S.J. (1990). On Intelligence…More or Less: A Bio-Ecological Treatise on Intellectual Development.

[5] Ceci S.J. (1990). On the relationship between microlevel and macrolevel processing efficiencies. Intelligence.

[6] Ferrer E., McArdle J.J., Shaywitz B.A., Holahan J.M., Marchione K., Shaywitz S.E. (2007). Longitudinal models of developmental dynamics between reading and cognition from childhood to adolescence. Dev. Psychol..

[7] Ferrer E., Shaywitz B.A., Holahan J.M., Marchione K., Shaywitz S.E. (2010). Uncoupling of reading and IQ over time: Empirical evidence for a definition of dyslexia. Psychol. Sci..

[8] Soares D.L., Lemos G.C., Primi R., Almeida L.S. (2015). The relationship between intelligence and academic achievement throughout middle school: The role of students’ prior academic performance. Learn. Individ. Differ..

[9] Reynolds M.R., Turek J.J. (2012). A dynamic developmental link between verbal comprehension-knowledge (Gc) and reading comprehension: Verbal comprehension-knowledge drives positive change in reading comprehension. J. Sch. Psychol..

[10] McGrew K.S., Flanagan D.P., Genshaft J.L., Harrison P.L. (1997). Analysis of the major intelligence batteries according to a proposed comprehensive Gf-Gc framework. Contemporary Intellectual Assessment: Theories, Tests, and Issues.

[11] McGrew K.S., Flanagan D.P. (1998). The Intelligence Test Desk Reference (ITDR): Gf-Gc Cross-Battery Assessment.

[12] Flanagan D.P., McGrew K.S., Ortiz S.O. (2000). The Wechsler Intelligence Scales and CHC Theory: A Contemporary Approach to Interpretation.

[13] Quinn J.M., Wagner R.K., Petscher Y., Lopez D. (2015). Developmental relations between vocabulary knowledge and reading comprehension: A latent change score modeling study. Child Dev..

[14] Primi R., Ferrao M.E., Almeida L.S. (2010). Fluid intelligence as a predictor of learning: A longitudinal multilevel approach applied to math. Learn. Individ. Differ..

[15] Campbell D.T., Stanley J.C. (1963). Experimental and Quasi-Experimental Designs for Research.

[16] Finkel S.E. (1995). Causal Analysis with Panel Data.

[17] Kenny D.A. (2005). Correlation and Causality.

[18] Crano W.D., Kenny D.A., Campbell D.T. (1972). Does intelligence cause achievement? A cross-lagged panel analysis. J. Educ. Psychol..

[19] Kellaghan T. (1973). Intelligence and achievement in a disadvantaged population: A cross-lagged panel analysis. Ir. J. Educ..

[20] Rogosa D. (1980). A critique of cross-lagged correlation. Psychol. Bull..

[21] Ritchie S.J., Bates T.C., Plomin R. (2015). Does learning to read improve intelligence? A longitudinal multivariate analysis in identical twins from age 7 to 16. Child Dev..

[22] Vitaro F., Brendgen M., Arseneault L. (2009). The discordant MZ-twin method: One step closer to the holy grail of causality. Int. J. Behav. Dev..

[23] Cole D.A., Preacher K.J. (2014). Manifest variable path analysis: Potentially serious and misleading consequences due to uncorrected measurement error. Psychol. Methods.

[24] Olson R.K., Keenan J.M., Byrne B., Samuelsson S. (2014). Why do children differ in their development of reading and related skills?. Sci. Stud. Read..

[25] Plomin R., Deary I.J. (2015). Genetics and intelligence differences: Five special findings. Mol. Psychiatry.

[26] Little T.D. (2013). Longitudinal Structural Equation Modeling.

[27] Little T.D., Preacher K.J., Card N.A., Selig J.P. (2007). New developments in latent variable panel analyses of longitudinal data. Int. J. Behav. Dev..

[28] Burkholder G.J., Harlow L.L. (2003). An illustration of a longitudinal cross-lagged design for larger structural equation models. Struct. Equ. Model..

[29] McArdle J.J. (2009). Latent variable modeling of differences and changes with longitudinal data. Annu. Rev. Psychol..

[30] Selig J.P., Little T.D., Laursen B., Little T.D., Card N.A. (2012). Autoregressive and cross-lagged panel analysis for longitudinal data. Handbook of Developmental Research Methods.

[31] Newsom J.T. (2015). Longitudinal Structural Equation Modeling: A Comprehensive Introduction.

[32] Geiser C. (2013). Data Analysis with Mplus.

[33] Wechsler D. (1991). Wechsler Intelligence Scale for Children—Third Edition.

[34] Watkins M.W., Lei P.-W., Canivez G.L. (2007). Psychometric intelligence and achievement: A cross-lagged panel analysis. Intelligence.

[35] Wechsler D. (2003). Wechsler Intelligence Scale for Children—Fourth Edition.

[36] Wechsler D. (2003). Wechsler Intelligence Scale for Children—Fourth Edition Technical and Interpretive Manual.

[37] Canivez G.L. (2014). Construct validity of the WISC-IV with a referred sample: Direct versus indirect hierarchical structures. Sch. Psychol. Q..

[38] Gomez R., Vance A., Watson S.D. (2016). Structure of the Wechsler Intelligence Scale for Children—Fourth Edition in a group of children with ADHD. Front. Psychol..

[39] Parkin J.R., Beaujean A.A. (2012). The effects of Wechsler Intelligence Scale for Children—Fourth Edition cognitive abilities on math achievement. J. Sch. Psychol..

[40] Salvia J., Ysseldyke J.E., Bolt S. (2010). Assessment in Special and Inclusive Education.

[41] Breaux K.C. (2009). Wechsler Individual Achievement Test-Third Edition Technical Manual.

[42] McGrew K.S., LaForte E.M., Schrank F.A. (2014). Woodcock-Johnson IV: Technical Manual.

[43] Muthén B.O., Muthén L.K. (2017). Mplus User’s Guide.

[44] Brown T.A. (2015). Confirmatory Factor Analysis for Applied Research.

[45] Giofrè D., Borella E., Mammarella I.C. (2017). The relationship between intelligence, working memory, academic self-esteem, and academic achievement. J. Cogn. Psychol..

[46] Alloway T.P., Alloway R.G. (2010). Investigating the predictive roles of working memory and IQ in academic attainment. J. Exp. Child Psychol..

[47] Beaujean A.A., Parkin J., Parker S. (2014). Comparing Cattell-Horn-Carroll factor models: Differences between bifactor and higher order factor models in predicting language achievement. Psychol. Assess..

[48] Hamaker E.L., Kuiper R.M., Grasman R.P. (2015). A critique of the cross-lagged panel model. Psychol. Methods.

[49] Calvin C.M., Fernandes C., Smith P., Visscher P.M., Deary I.J. (2010). Sex, intelligence and educational achievement in a national cohort of over 175,000 11-year-old schoolchildren in England. Intelligence.

[50] Cooper C. (2015). Intelligence and Human Abilities: Structure, Origins and Applications.

[51] Deary I.J. (2001). Intelligence: A Very Short Introduction.

[52] Duckworth A.L., Quinn P.D., Tsukayama E. (2012). What No Child Left Behind leaves behind: The roles of IQ and self-control in predicting standardized achievement test scores and report card grades. J. Educ. Psychol..

[53] Gottfredson L.S., Prifitera A., Saklofske D.H., Weiss L.G. (2008). Of what value is intelligence? In WISC-IV Clinical Assessment and Intervention.

[54] Gottfredson L.S. (2016). Hans Eysenck’s theory of intelligence, and what it reveals about him. Personal. Individ. Differ..

[55] Kuncel N.R., Hezlett S.A. (2010). Fact and fiction in cognitive ability testing for admissions and hiring decisions. Curr. Dir. Psychol. Sci..

[56] Lubinski D. (2004). Introduction to the special section on cognitive abilities: 100 years after Spearman’s (1904) “general intelligence, objectively determined and measured”. J. Personal. Soc. Psychol..

[57] Meldrum R.C., Petkovsek M.A., Boutwell B.B., Young J.T.N. (2017). Reassessing the relationship between general intelligence and self-control in childhood. Intelligence.

[58] Ritchie S. (2015). Intelligence: All that Matters.

[59] Sternberg R.J., Kaufman S.B. (2011). The Cambridge Handbook of Intelligence.

[60] Macintosh N.J. (2011). IQ and Human Intelligence.

[61] Mulaik S.A. (2009). Linear Causal Modeling with Structural Equations.

[62] Shaddish W.R., Cook T.D., Campbell D.T. (2002). Experimental and Quasi-Experimental Designs for Generalized Causal Inference.

[63] Sternberg R.J., Gigorenko E., Bundy D.A. (2001). The predictive value of IQ. Merrill-Palmer Q..

[64] Schneider W., Niklas F., Schmiedeler S. (2014). Intellectual development from early childhood to early adulthood: The impact of early IQ differences on stability and change over time. Learn. Individ. Differ..

[65] Canivez G.L., Watkins M.W., James T., Good R., James K. (2014). Incremental validity of WISC-IV^UK^ factor index scores with a referred Irish sample: Predicting performance on the WIAT-II^UK^. Br. J. Educ. Psychol..

[66] Glutting J.J., Watkins M.W., Konold T.R., McDermott P.A. (2006). Distinctions without a difference: The utility of observed versus latent factors from the WISC-IV in estimating reading and math achievement on the WIAT-II. J. Spec. Educ..

[67] Schneider W., Niklas F. (2017). Intelligence and verbal short-term memory/working memory: Their interrelationships from childhood to young adulthood and their impact on academic achievement. J. Intell..

[68] Beebee H., Hitchcock C., Menzies P. (2009). Oxford Handbook of Causation.

[69] Imbens G.W., Rubin D.B. (2015). Causal Inference for Statistics, Social, and Biomedical Sciences: An Introduction.

[70] Pearl J., Glymour M., Jewell N.P. (2016). Causal Inference in Statistics: A Primer.

[71] Shadish W.R. (2010). Campbell and Rubin: A primer and comparison of their approaches to causal inference in field settings. Psychol. Methods.

[72] Watkins M.W., Glutting J.J. (2000). Incremental validity of WISC-III profile elevation, scatter, and shape information for predicting reading and math achievement. Psychol. Assess..

